# Studies about the Dietary Impact on “Free” Glycation Compounds in Human Saliva

**DOI:** 10.3390/foods11142112

**Published:** 2022-07-15

**Authors:** Friederike Manig, Michael Hellwig, Franziska Pietz, Thomas Henle

**Affiliations:** 1 Chair of Food Chemistry, Technische Universität Dresden, D-01062 Dresden, Germany; fpietz@web.de; 2 Chair of Special Food Chemistry, Technische Universität Dresden, D-01062 Dresden, Germany

**Keywords:** Maillard reaction, glycation, saliva, metabolic transit, Advanced Glycation Endproducts (AGEs), biomarker, humans

## Abstract

Glycation reactions play a key role in post-translational modifications of amino acids in food proteins. Questions have arisen about a possible pathophysiological role of dietary glycation compounds. Several studies assessed the metabolic fate of dietary glycation compounds into blood and urine, but studies about saliva are rare. We investigated here the dietary impact on salivary concentrations of the individual Maillard reaction products (MRPs) N-ε-fructosyllysine, N-ε-carboxymethyllysine (CML), N-ε-carboxyethyllysine (CEL), pyrraline (Pyr), and methylglyoxal-derived hydroimidazolone 1 (MG-H1). Quantitation was performed using stable isotope dilution analysis (LC-MS/MS). We describe here, that a low MRP diet causes a significant lowering of salivary levels of Pyr from 1.9 ± 0.4 ng/mL to below the LOD and MG-H1 from 2.5 ± 1.5 ng/mL to 0.7 ± 1.8 ng/mL. An impact on the salivary protein fraction was not observed. Furthermore, salivary Pyr and MG-H1 levels are modified in a time-dependent manner after a dietary intervention containing 1.2 mg Pyr and 4.7 mg MG-H1. An increase in mean salivary concentrations to 1.4 ng/mL Pyr and 4.2 ng/mL MG-H1 was observed within 30–210 min. In conclusion, saliva may be a useful tool for monitoring glycation compound levels by using Pyr and MG-H1 as biomarkers for intake of heated food.

## 1. Introduction

A plenty of studies are dealing with the question of the physiological impact of dietary glycation compounds on health as controversial issue. Maillard reaction products (MRP) arise during heating, storage, and processing in the course of the Maillard reaction between reducing sugars and amino compounds. The first stage of the Maillard reaction is characterized by the formation of Amadori compounds such as N-ε-fructosyllysine (FruLys). Amadori compounds may be degraded during further reactions to form dicarbonyl compounds. The final stage of the reaction leads to the formation of Advanced Glycation Endproducts (AGEs) such as N-ε-carboxymethyllysine (CML), N-ε-carboxyethyllysine (CEL), methylglyoxal-derived hydroimidazolone 1 (MG-H1), and pyrraline (Pyr) [1]. Beside the occurrence of free MRPs, the modification of protein side chains is critical for lysine and arginine. Despite some specific diets such as raw-foodism, food is mostly heated or stored before ingestion. As a result, nutritional and sensory characteristics are altered. Concentrations in food cover a wide range, depending on the extent of the Maillard reaction, where Amadori compounds are of higher quantitative relevance. Appropriate databases about concentrations in food can be found in the literature or online (AGE database [2] and Scheijen et al. 2016 [3]). The quantitative information about individual glycation compounds allows an estimation of the daily intake. An overview about the ingested daily amount of specific MRPs is given in Table 1. However, the daily intake of Amadori compounds is much higher than that of AGEs owing to their higher prevalence in food [4]. After ingestion, glycated proteins may undergo proteolytic cleavage [5,6,7]. Table 1 summarizes MRP concentrations analyzed in some body fluids.

Glycation compounds formed endogenously are discussed to be of clinical relevance. Physiological consequences may result when glycation occurs in the human body by physiological levels of dicarbonyl compounds and sugars and may increase during hyperglycemia and additionally be altered by oxidative stress etc. Beside diabetic complications it was shown in this context that mice receiving a high glucose or fructose load develop sugar-specific glycation patterns in the liver tissue [15,16]. By focusing on the molecular level, it remains clear that effects of dietary and endogenous MRPs have to be strongly differentiated. The metabolic transit of ingested MRPs from food to the circulation strongly depends on the individual chemical nature of the MRP. Flux studies with different MRPs on Caco-2 cells imply a better absorption of peptide-bound MRPs by gastrointestinal peptide transporters [17]. However, glycation compounds may undergo metabolic transit through the body. Several studies are dealing with the quantification of glycation compounds in various body fluids after metabolic transit [8,18,19,20], but studies about salivary glycation compounds are rare.

Saliva consists of 99.5% water and 0.1–0.3% proteins—among them more than 1200 different proteins and a great variety of minor compounds [21,22]. Saliva mirrors the blood levels of compounds such as caffeine, hormones, and amino acids with concentration gradient from 1:1 to 1:1000 [23]. The transfer from blood to saliva takes place by ultrafiltration, diffusion [24], and active transport mechanisms [21]. Prognostic potential of saliva for early disease detection includes e.g., amino acid profile for early cancer diagnosis [25], protein analytics for early diagnosis of heart attack [26], diagnosis and monitoring of glucose in type 2 diabetes [27], and others [28]. A putative metabolic transit of some glycation compounds from blood to saliva was recently expected by our group [29]. The aim of the current study was to investigate the dietary impact on “free” and protein-bound glycation compounds in saliva. In the current study, we tested different dietary styles to assess and substantiate the dietary impact on salivary “free” MRP concentrations during a five-day testing. Furthermore, we provide a deeper insight into the time course of metabolic transits of a defined amount of ingested food MRPs to saliva.

## 2. Materials and Methods

### 2.1. Chemicals

Acetonitrile (LC-MS grade) and methanol (HPLC grade) were obtained from VWR Prolabo (Darmstadt, Germany). Freshly double distilled water (Bi 18E double distillation system, QCS, Maintal, Germany) was used for solvents production for LC-MS analysis. Nonafluoropentanoic acid (NFPA) was from Sigma-Aldrich (Steinheim, Germany). Reference material for calibration of MRPs was synthesized in our laboratory as described before: N-ε-fructosyllysine [30], pyrraline [31,32], CML [17], CEL [17], and MG-H1 [17]. Stable isotope labelled internal standards for HPLC-MS/MS analysis were synthesized similarly but using [^13^C_6_,^15^N_2_]lysine ([^13^C_6_,^15^N_2_]Pyr) and [^13^C_6_]arginine ([^13^C_6_]MG-H1) instead of the unlabeled compounds. The synthesis of [^13^C_3_]CEL was described recently [29]. [^2^H_2_]CML was purchased from PolyPeptide (Strasbourg, France), [^13^C_6_,^15^N_2_]lysine from Campro (Berlin, Germany), and [^13^C_6_]arginine from Eurisotop (Saarbrücken, Germany). Enzymatic hydrolysis was performed by using pronase E (Merck, Darmstadt, Germany) and pepsin, aminopeptidase and prolidase (all from Sigma-Aldrich, Steinheim, Germany).

### 2.2. Food Samples

Food samples analyzed during this study were obtained from local supermarkets in Dresden, Germany. The content of protein-bound MRPs was analyzed after enzymatic hydrolysis. Protein hydrolysates were prepared as described earlier [33]. In brief, homogenized food samples were placed in glass flasks according to a protein amount of 2–3 mg per sample and incubated in 1 mL 0.02 M HCl and 50 µL pepsin (1 U/sample) at 37 °C. After 24 h, 250 µL Tris-HCl buffer (pH 8.2) and 50 µL of a pronase E solution (400 U/sample) were added to each sample. After further 24 h, further 2 µL aminopeptidase solution (0.3 U/sample) and 20 µL prolidase solution (2 U/sample) were added. Further sample preparation steps included lyophilization, resuspension in 1 mL bidistilled water, deproteinization, defatting, and cleaning-up with solid phase extraction as described earlier [14].

### 2.3. Study Design

The study has been approved by the Ethics Committee of Technische Universität Dresden, Germany (reference: AZ 439112017). Written consent was obtained from each study participant. All of the study participants were of normal weight and healthy. The first part of the study, a pilot study, included six subjects (one subject was dropped out due to its food log (*n* = 5, gender: 3 female, 2 male, age 22–28, mean age 26.4, median age 27). The subject cohort in the second part of the study included *n* = 17 subjects (12 female, 5 male, age 22–38, mean age 25.0, median age 23.0). Subjects with severe metabolic diseases were excluded in order to focus on the dietary impact.

First part of the study (crossover design): To investigate a putative dietary impact on salivary MRP levels, the subjects were asked to eat a diet low in MRPs (mainly raw food: vegetables, fruits, unroasted nuts) for four days. To assess a putative increase in salivary MRP levels, subjects were asked to eat as usual on day five. The week after, the subjects were asked to eat as usual but to add food high in MRP content (mainly sweet and salty snacks such as crisps and pretzel sticks as well as other baked goods).

Second part of the study (intervention study): The subjects were asked to eat a diet low in MRP content as it was described in the first part of the study for two or three days, respectively. In the morning of the third day, an intervention meal was provided to the subjects consisting of 70 g cookies, 60 g salt sticks, and 30 g crisps. The food for intervention was chosen for high MRP content in order to potentially evoke an increase in salivary MRP concentrations.

### 2.4. Sampling

Fasting saliva collection was performed before breakfast with Salivettes (Sarstedt, Germany) and during the day with the following method. The manufacturer’s protocol for sampling was adapted to use Salivettes for saliva collection without stimulation. The subjects were obliged to brush the teeth without tooth paste, rinse the mouth with water, and wait for five minutes. Salivettes were placed in the middle of the tongue. Tongue movement should be avoided within three minutes of saliva collection.

### 2.5. Preparation of Saliva Samples for MRP Detection

Samples were prepared for analysis as described before [29]. In brief, Salivettes were centrifuged (2 min, 2500× *g*) and the collected saliva transferred in a tube. Internal standard solution (10 µL) was added to 300–500 µL of saliva, followed by a deproteinization using 500 µL icecold acetonitril/methanol (70/30, *v*/*v*). The tubes were centrifuged (10,000× *g*, 10 min) and the supernatant was transferred in a new tube for evaporation to dryness under a stream of nitrogen. The remnant in the tubes was redissolved in 20 mM NFPA. Samples were usually analyzed in duplicate. Bidistilled water was used as basis for blanks. Blanks were treated as samples during processing. The external calibration of CML, CEL, MG-H1, Pyr, FruLys as well as Arg and Lys with each of the corresponding isotopologue internal standard was treated like samples including evaporation to dryness under nitrogen and redisssolving in 20 mM NFPA. The calibration curve for MRP was linear between 0.003 and 0.15 µg/mL and for amino acids between 0.1 and 150 µg/mL.

For the analysis of salivary protein fraction, 500 µL ice-cold acetonitril/methanol (70/30, *v*/*v*) was added to 500 µL of saliva. The protein fraction was earned after centrifugation (10,000× *g*, 10 min) and enzymatically hydrolyzed as described above for the food samples.

### 2.6. High-Pressure Liquid Chromatography with Tandem Mass Spectrometric Detection (HPLC-MS/MS)

“Free” MRPs were quantitated as described before [29] on an HPLC-MS/MS system consisting of a binary pump (G1312A), an online degasser (G1379B), an autosampler (G1329A), a column thermostat (G1316A), a diode array detector (G1315D), and a triple-quadrupole mass spectrometer (G6410A; all from Agilent Technologies, Böblingen, Germany). At the ESI source, nitrogen was utilized as the nebulizing gas with a gas flow of 11 L/min, a gas temperature of 350 °C, and a nebulizer pressure of 35 psi. The capillary voltage was at 4000 V. Samples were run on a Phenomenex Kinetex C-18 column (50 × 2.1 mm, 1.7 µm, 100 Å; from Phenomenex LTD, Aschaffenburg, Germany) and an injection volume of 5 µL was used for chromatographic separation. Solvent A consisted of 10 mM NFPA in water, solvent B was 10 mM NFPA in acetonitrile. A gradient (0 min, 5% B; 10 min, 32% B; 11 min, 85% B; 14 min, 85% B; 15 min, 5% B) with a flow rate of 0.25 mL/min was used. The software Mass Hunter B.02.00 (Agilent) was used for data acquisition. Quantitation was performed by using MRM transitions and validation parameters published earlier [29]. Samples were processed and analyzed in duplicate. For calculations, the LOD concentration was used if peaks were detected below the LOD, if no peaks were detected, peak areas were set “0”.

### 2.7. Statistics

Statistical analyses (correlation analysis: Spearman, test for normal distribution: Kolmogorov–Smirnov, Levene’s test for homogeneity of variances, paired Student’s t-test and ANOVA) were performed by using the software OriginPro 2015G and 2019 (OriginLab Corporation, Northampton, MA, USA). The calculation of the area under the curve was performed with OriginPro 2015G by using the respective AUC tool. Box plots were plotted with the same software. The box contains 50% of data and includes the mean of the data (demonstrated by the square in the box) and the median (demonstrated by the line in the box). The length of the box, the interquartile range, is equivalent with the variation of the data. The whiskers within plots show a range of 1.5-fold values of the interquartile range. Outliers are indicated by a rhombus. Significances are demonstrated according to the respective significance levels with *p* < 0.05 (*), *p* < 0.01 (**), and *p* < 0.001 (***).

## 3. Results

In the present study, we examined the impact of dietary MRPs on salivary MRP concentrations and strengthen the role of Pyr, and with reservations, MG-H1 as putative salivary biomarkers to differentiate between the exogenous and the endogenous MRP pool. The possibility of frequent sampling is one of the major advantages of saliva as sample matrix. Due to the non-invasive sampling technique, we could follow the course of MRPs after an intervention meal with intervals of 2 h.

### 3.1. Individual MRP Concentrations in Saliva Can Be Altered by Dietary Changes

The results of our earlier pilot study suggested a transit of specific dietary MRPs, namely Pyr and MG-H1, from the food to saliva [29]. To assess the impact of dietary MRP intake on salivary MRP concentrations and to evaluate and substantiate a putative metabolic transit, we performed a study consisting of two parts. During the first part, the subjects were asked to eat a diet low in MRPs for four days. Subjects were allowed to eat e.g., raw fruits and vegetables, nuts (except cashews), native oils and raw salmon and raw meat. On the fifth day, the subjects were asked to eat as usual. The week after, the food of the subjects consisted of a usual diet supplemented with food high in MRP concentrations.

**Figure 1:**
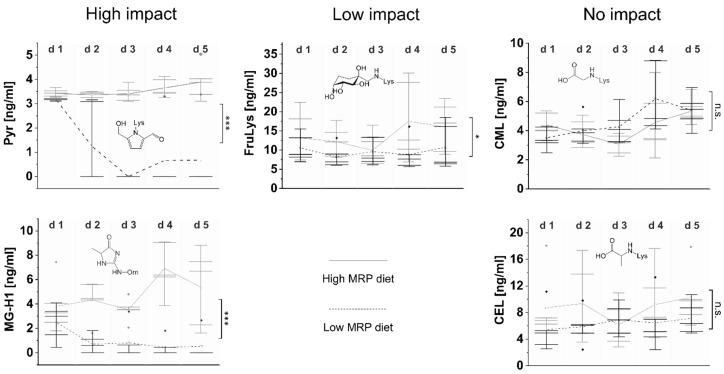
Impact of dietary MRP intake on salivary MRP concentrations, analyzed during a crossover-study with low and high MRP intake (grey: high MRP intake, black: low MRP intake). Shown are boxplots of salivary concentrations of individual MRPs in the morning of five consecutive days of five subjects with sampling at 7 am. Outliers are indicated by a rhombus. Connected lines indicate data points of mean salivary MRP concentrations (low MRP diet: dashed line versus high MRP diet: solid line). The statistical analysis shown in the graph is related to the low versus high MRP diet curves. Significance: *** *p* < 0.001, * *p* < 0.05, n.s.—not significant.

**Figure 2:**
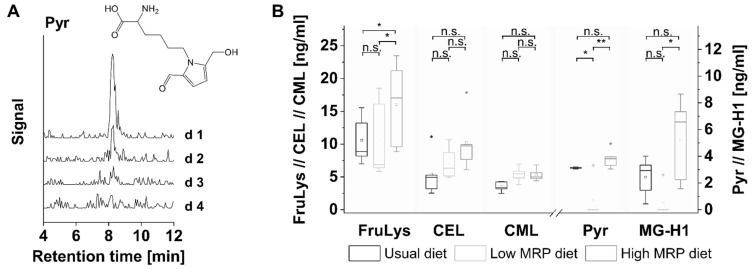
(**A**) LC-MS/MS chromatogram of pyrraline (Pyr) in saliva samples of one subject on four consecutive days during low MRP diet; (**B**) comparison of dietary styles: boxplot of salivary MRP concentrations of five subjects during usual diet, on day 5 of the low MRP diet and on day 5 of the high MRP diet. The box contains 50% of data and includes the mean of the data (demonstrated by the square in the box) and the median (demonstrated by the line in the box). Outliers are indicated by a rhombus. Significance:, ** *p* < 0.01, * *p* < 0.05, n.s.—not significant.

The morning salivary concentrations of the individual AGEs CML, CEL, MG-H1, Pyr and the Amadori compound FruLys during low versus high MRP diet and the corresponding statistical analysis are provided in Figure 1 with statistical calculations in Table 2. The term “d1 low” represents the starting conditions after the usual diet. During the first two to three days of the low MRP diet, a strong decrease in salivary MG-H1 and Pyr levels was observed. Pyr was lowered within two days of low MRP diet from 1.9 ± 0.4 ng/mL to below the limit of detection. MG-H1 was lowered from 2.5 ± 1.5 ng/mL to 0.7 ± 1.8 ng/mL with 4 of 6 subjects < LOD. The corresponding peaks for Pyr as measured with LC-MS/MS are shown in Figure 2A. Mean salivary levels of Pyr and MG-H1 remained below LOD during low MRP diet. The slight increase of Pyr on day 4/5 (Figure 1) can be attributed to the salivary Pyr concentrations of one of the subjects. As the same subjects showed a salivary MG-H1 concentration below the LOD, the subjects’ keeping of low MRP diet rules can be expected. After completion of the low MRP diet and starting a conventional diet, we observed a clear increase in salivary Pyr and MG-H1 concentrations which remained high during the time with high MRP intake (please see Figure S1). It seems that the decrease of Pyr and MG-H1 during the wash-out phase of two to three days took much longer than the increase in salivary concentrations when the subjects were allowed to eat as usual again (Figure S1). The course of salivary Pyr and MG-H1 concentration graphs indicates a significant difference between low and high MRP intake, thereby evoking clear evidence for a dietary impact on salivary MG-H1 and Pyr concentrations. The statistical analysis was performed by using the data obtained during the low MRP diet and the high MRP diet. Beside AGEs, FruLys as an Amadori compound was investigated. The slight differences in comparison of the graphs during low and high MRP intake are reflected by the statistical analysis. The time course of FruLys graphs indicate not a high, but still significant difference between both forms of dietary intervention. In contrast to the low MRP diet, the high MRP diet did not evoke significant differences in comparison to the usual diet with an exception for FruLys (Figure 2B). We conclude here, that MRP concentrations above a certain threshold may not be attributed to the dietary intake.

For salivary free CML and CEL, no pronounced difference between low and high MRP intake was observed. In our study based on saliva as a body fluid, both carboxyalkyl amino acids (CML and CEL) were not altered by dietary exposure.

### 3.2. A High MRP Intervention Meal Causes An Increase of Selected Salivary MRP Concentrations after Low MRP Diet

Due to the fast reversibility of salivary MG-H1 and Pyr wash-out within hours (Figure 1), our aim was to investigate the time course of increasing concentrations subsequent to an intervention meal. During the second part of the study, 17 subjects were asked to eat a low MRP diet for three days as well as an intervention meal in the morning of d 3. The composition of the intervention meal is listed in Table 3.

**Figure 3:**
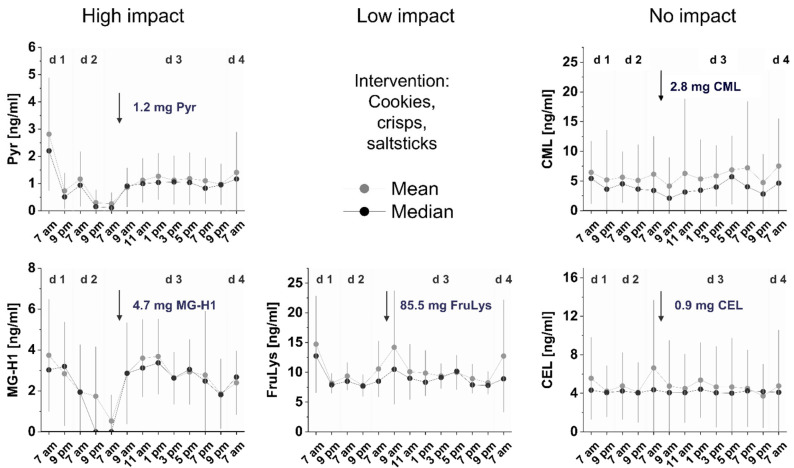
Impact of high MRP intervention meal on salivary MRP concentrations during a three-day low MRP diet. The arrows indicate the timepoint of intervention according to Table 3. Data indicate mean salivary MRP concentrations of *n =* 17 subjects with one-sided SD.

Again, no alterations of salivary concentrations before or after intervention were found for CML and CEL for most of the subjects. In contrast, for Pyr and MG-H1, respectively, again a reproducible decrease in salivary concentrations after 2 d of low MRP diet was found. This observation is in conclusion with data from the first part of the study. Data for salivary mean and median MRP concentrations are shown in Figure 3. After ingestion of the intervention meal, MG-H1 concentrations reached the level of 4.2 ± 2.2 ng/mL similar to those before the low MRP diet within three to five hours, depending on the subject. The graphs for two subjects with a pronounced lowering during low MRP diet and the remarkable increase after intervention is shown in Figure S2A. For Pyr, the mean concentrations reached 1.4 ± 0.9 ng/mL, corresponding to ~50% of the starting point. In the first part of the study, it was found that salivary FruLys concentrations are also slightly modulated by the amount of dietary in- and uptake. However, the single intervention meal did not cause a significant increase in salivary FruLys concentrations in most subjects. In three subjects, we could observe a considerable increase. The graphs for each of the subjects is given in Figure S2B. In general, the high interindividual differences in the salivary concentrations may result from differences in the individual metabolic handling of MRPs during absorption in each subject but may also depend on transport mechanisms from blood to saliva. A reabsorption of salivary Pyr after deglutition remains unlikely since Pyr is not absorbed as free amino acid [17]. The single graphs for each of the subjects are given in Figures S2B and S3.

Due to a longer wash-out time as expected from the first part of the study, it would have been worthy to prolong the low MRP diet before intervention. A higher MRP load during intervention would have been also precious but would have been hard to perform for the subjects which already suffered from the intervention meal—the acceptance of the intervention meal by the subjects was very low.

### 3.3. Pyrraline: A Marker for the Uptake of Heated Food and the Individual Metabolic Transit of Dietary MRPs?

For FruLys and Pyr, we could observe clear interindividual differences in some subjects by showing higher increases in salivary concentrations after exogenous MRP load during intervention. We focus in this section on Pyr since the low MRP diet led to non-detectable Pyr peaks in some subjects.

**Figure 4:**
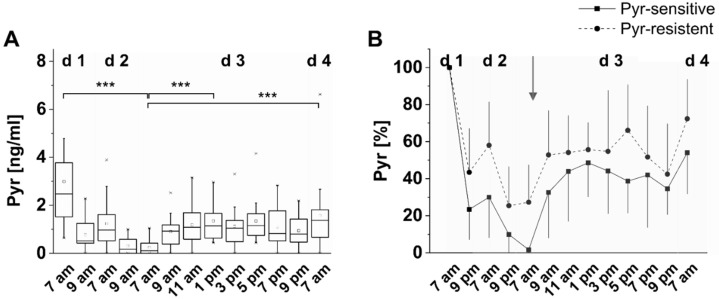
Interindividual differences in the metabolic transit of 1.2 mg dietary Pyr from food to saliva. (**A**) Boxplot of salivary Pyr concentrations after a high MRP intervention meal during a three-day low MRP diet of 17 subjects. The box contains 50% of data and includes the mean of the data (demonstrated by the square in the box) and the median (demonstrated by the line in the box). The length of the box, the interquartile range, is equivalent with the variation of the data. The whiskers within plots show a range of 1.5-fold values of the interquartile range. Outliers are indicated by an asterisk. Significance: *** *p* < 0.001. (**B**) classification of subjects corresponding to the degree of decrease of salivary Pyr concentration during low MRP diet in two subgroups of “Pyr-sensitive metabolizers”(squares) and “Pyr-non-sensitive metabolizers”(dots). Shown are the mean values with one-sided standard deviation. The arrow indicates the time of intervention.

Interindividual differences in salivary concentrations are pronounced to a different degree during the study time and are shown exemplarily for Pyr in Figure 4A for all of the *n* = 17 subjects. Food as usual (d1 at 7 am) leads to a higher degree of variation in salivary Pyr concentrations among subjects whereas differences are getting smaller during low MRP diet (d3 at 7 am). Most probably, this may not only be due to the amount of ingested Pyr. During data analysis, a particularity among subjects occurred: Salivary Pyr concentrations had fallen below the limit of detection in *n* = 5 subjects, but not in *n* = 12 others. Figure 4B presents a grouping of these “Pyr sensitive” and “Pyr resistant” individuals which show a lower decrease before and only a slight increase in salivary Pyr concentrations after the intervention meal. Beside the assumption that the metabolic transit of Pyr differs individually, this observation may result from a higher sensitivity toward MRP absorption and elimination or permeability of the gut barrier. An association to (patho-)physiological states such as a leaky gut or kidney diseases may be also worth considering but was not described by the subjects. However, kidney diseases may not only result in higher concentrations but also in a longer half-life in the body and remain to be investigated during further studies. However, even if there is consent on Pyr as biomarker for consumption of heated food (see Section 4), endogenous sources may be not completely excluded from the discussion.

### 3.4. Short-Term Low MRP Diet Does Not Alter the Glycation of the Salivary Protein Fraction

**Figure 5:**
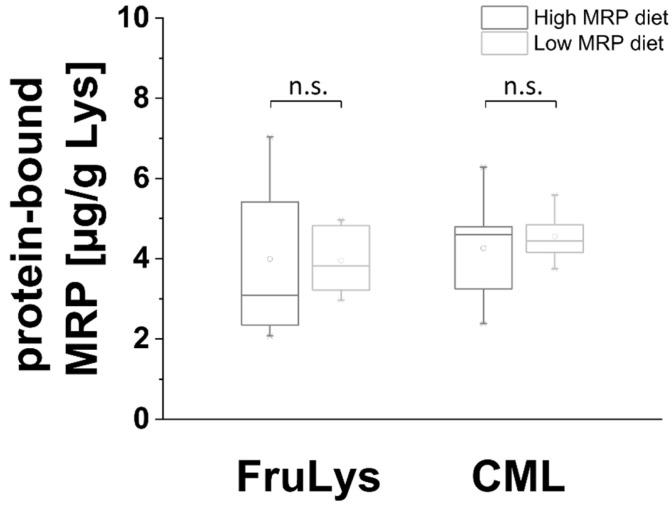
Boxplot of protein-bound FruLys and CML concentrations in the salivary protein fraction of five subjects during low or high MRP diet (sampling on d 3). The box contains 50% of data and includes the mean of the data (demonstrated by the dot in the box) and the median (demonstrated by the line in the box). Significance: n.s.—not significant.

A further aim was to determine whether differences in the dietary MRP load also lead to alterations in the salivary protein fraction. For this aim, the salivary protein fraction was isolated after centrifugation. Only FruLys and CML could be observed in salivary protein fractions after enzymatic hydrolysis followed by LC-MS/MS analysis. The results are shown in a boxplot in Figure 5. No significant differences were observed for protein-bound FruLys or CML during low vs. high MRP diet.

## 4. Discussion

The possible pathophysiological role of dietary MRPs has been under critical discussion for years. There is thus the need for clarification whether or to what extent dietary MRPs contribute to the endogenous pool of glycation compounds. A couple of studies were published investigating the metabolic transit of dietary MRPs to blood or urine. To the best of our knowledge, saliva was not yet investigated in systematic studies about the metabolic transit of individual glycation compounds. On the basis of a former pilot study [29], the present study was performed to analyze associations between the dietary intake of AGEs and the impact on salivary AGE concentrations. Furthermore, an estimation of kinetics from food to saliva is included and for the first time, the salivary protein fraction was analyzed for its concentration of individual glycated amino acids by mass-spectrometric techniques in order to observe possible alterations of the glycation pattern after dietary manipulation.

A highly significant dietary impact on salivary AGE concentrations was found for Pyr and MG-H1 which is in conclusion with other studies analyzing the metabolic transit to bovine and human milk [14] or, at least partly, plasma and urine in the CODAM study [8]. In the CODAM study, an association between dietary intake and plasma and urine levels of MG-H1, CML, and CEL was observed. In the present study, salivary CML and CEL levels were not found to reflect the dietary intake. However, our study was focusing on a short-term intervention and not on long-term nutrition. Still, one limitation in our study is the style of a pilot study with a comparably small cohort. Therefore, the results are not representative due to the limited statistical power, but may show a direction for further studies. Pathophysiological conditions were also not part of the study and the metabolic transit of MRPs may be altered, particularly in renal disease. Furthermore, the time point of the intervention meal after low MRP diet was estimated from the first part of the study. It seems that two days of low MRP diet would be sufficient for lowering the salivary Pyr and MG-H1 concentrations below the LOD. The analysis of a bigger cohort revealed that salivary concentrations had not been lowered below the LOD at this time point for every participant. Even if we have not tested whether a complete Pyr washout in saliva below the LOD is possible for everyone, a buffer of one more day with low MRP diet may have been more practical.

The results of the study may do not have direct nutritional consequences. However, this study provides further evidence for a mainly dietary source of Pyr as concluded also from other authors. Pyr is discussed as a marker for dietary MRP uptake and thus originate exclusively from the diet [18,34,35]. Furthermore, beside Pyr, MG-H1 seems to be affected as well and may act as biomarker for the intake of heated food.

Interestingly, the time between intake of the intervention meal and the increase of salivary Pyr and MG-H1 concentrations is quite short. However, this is in consent with a study investigating D-amino acids in saliva: Nagata et al., found D-alanine and D-glutamate to be detectable in saliva 30 min after the ingestion of a meal [36]. Moreover, the short time interval of 1 to 3 h between the intervention meal and the increase in salivary MG-H1 and Pyr concentrations seems to be plausible in relation to amino acid and peptide absorption studies in plasma [37]. Since saliva is a kind of plasma filtrate, a quick influx of dietary components to plasma may lead to the assumption of a quick influx to saliva as well.

To estimate the amount of ingested MG-H1 and Pyr recovered in saliva, we calculated the area under the curve from the timepoint of the intervention until the next morning (Pyr 25.2 ng/24 h; 21.6 ng/24 h MG-H1) by using the AUC tool in OriginPro 2015G. The daily saliva production of healthy adults is about 500–1500 mL [21]. In relation to the intake (Table 3), we can estimate a recovery between 1.3–3.8% Pyr and 0.7–2.0% MG-H1 in saliva. A more comprehensive study may be useful to define a kind of “glycation index” to observe the increase after ingestion of certain food items.

ARPs such as FruLys are ingested with food in daily amounts of up to 1000 mg [9], relatively higher in comparison to Pyr, of which 20–40 mg are ingested daily [10]. The ratio of FruLys to Pyr taken up by food is thus about 16 and 980, depending on the food item. The ratio of FruLys to Pyr in saliva ranges from 3.3–36.3 in our samples, leading to the assumption that alimentary FruLys is transferred with a much smaller percentage into saliva than Pyr. Remarkably, salivary FruLys levels exceed Pyr levels, leading to the assumption of a recovery in saliva in a dose-dependent manner as suggested in the older literature for urine [18,20,38]. In contrast to lysine, FruLys does not underlie active transport mechanisms but diffusion whereby N^ε^-FruLys is better absorbed than N^α^-FruLys [20,39]. The authors demonstrated that 60–80% of the ingested free FruLys and 3–10% protein-bound FruLys can be recovered in the urine of humans and rats. It was demonstrated by a transwell Caco-2 cell culture model system that 10 mM FruLys reduces the lysine absorption about 80% und thus interacts with lysine transporter, but this does not result in a considerable absorption of FruLys [40].

We conclude here that salivary MG-H1 and Pyr concentrations do depend on dietary intake, but are not capable of leading to significant increase during normal food ingestion. Higher salivary concentration changes, especially of CEL and CML which were not altered by dietary changes, may theoretically arise during pathophysiological conditions.

## Figures and Tables

**Table 1:** Estimated daily intake of Maillard reaction compounds from food and concentrations in different human body fluids.

FruLys	CEL	CML	Pyr	MG-H1	Reference
Estimated or calculated daily intake [mg]					
	2.3 ± 0.8	3.1 ± 1.0		21.7 ± 6.7	[8]
500–1200					[9]
			20–40		[10]
Plasma free MRP [ng/mL]					
	9.8	15.9		27.6	[8] *
	8.1–12.6	12.2–20.2		19.6–39.4	
≤43.2	7.6 ± 3.1	4.7 ± 1.6		25.1 ± 10.5	[11]
	7.8–12.0	12.4–20.4		19.2–39.4	[12]
Serum free MRP [ng/mL]					
	9.8 ± 2.2	15.1 ± 4.2		17.5 ± 8.2	[13]
Human milk free MRP [ng/mL]					
17.7–32.6		12.0–23.1	10.4–56.9	4.8–18.0	[14]

* Calculated from nmol/mL.

**Table 2:** Statistical significance (paired t-test) during high versus low MRP diet with the significance levels *** *p* < 0.001, ** *p* < 0.01, * *p* < 0.05, n.s.—not significant. The term “d1 low” represents the starting conditions after the usual diet.

Versus	d 1 low	d 1 low	d 1 low	d 1 low	d 1 low	d 2 low	d 3 low	d 4 low	d 5 low	d 1 low
	d 2 low	d 3 low	d 4 low	d 5 low	d 1 high	d 2 high	d 3 high	d 4 high	d 5 high	d 5 high
Pyr	n.s.	***	*	*	n.s.	*	***	**	**	n.s.
MG-H1	n.s.	n.s.	n.s.	n.s.	n.s.	**	*	**	*	n.s.

**Table 3:** MRP content of the intervention meal, food analysis was performed using LC-MS/MS analysis after enzymatic hydrolysis.

Food	FruLys (mg)	CML (mg)	CEL (mg)	MG-H1 (mg)	Pyr (mg)
Cookies (70 g)	77.0	2.1	0.5	1.3	0.4
Crisps (30 g)	3.2	0.4	0.1	0.1	<0.1
Salt Sticks (60 g)	5.0	0.3	0.3	3.3	0.8
Cream Cheese (17 g)	<0.1 mg/100 g or n.d.				
Sum	85.2	2.8	0.9	4.7	1.2

## Data Availability

The data presented in this study are available in the Supplementary File (Tables S1–S3).

## Supplementary Materials

The following supporting information can be downloaded at: https://www.mdpi.com/article/10.3390/foods11142112/s1, Figure S1: Impact of dietary MRP intake on salivary Pyr (A) and MG-H1 (B) concentrations; Figure S2: Impact of a high MRP intervention meal on salivary (A) MG-H1 and (B) FruLys concentrations during a three-days low MRP diet; Figure S3: Impact of a high MRP intervention meal on salivary MRP concentrations during a three-days low MRP diet; Table S1: Raw data of Figure 1: Impact of dietary intake on salivary MRP concentration; Table S2: Raw data of the intervention study (Figure 3, Figure 4, Figure S2 and Figure S3); Table S3: Raw data of Figure 5: Concentrations of protein-bound FruLys and CML in the salivary protein fraction.
