# Supplementary material for: Studies about the Dietary Impact on “Free” Glycation Compounds in Human Saliva
Source: Foods. 2022 Jul 15;11(14):2112. doi: 10.3390/foods11142112 (PMC9324897; doi:10.3390/foods11142112)
Supplement: Supplementary file 1 [file foods-11-02112-s001.zip › foods-1784266-supplementary.pdf]

Supplementary materials

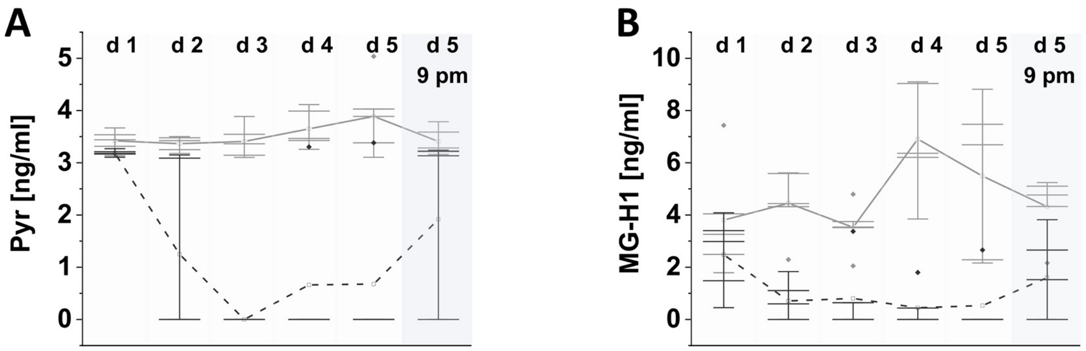

Figure S1. Impact of dietary MRP intake on salivary Pyr (**A**) and MG-H1 (**B**) concentrations, analyzed during a crossover-study with low and high MRP intake (grey: high MRP intake, black: low MRP intake). Shown are boxplots of salivary concentrations of individual MRPs in the morning of 5 consecutive days of five subjects with sampling at 7 am as well as the onset of eating the subjects' usual diet after four days of low MRP diet (d5 at 9 pm). Connected lines indicate data points of mean salivary MRP concentrations (low MRP diet: dashed black line versus high MRP diet: solid grey line). Outliers are indicated by a rhombus.

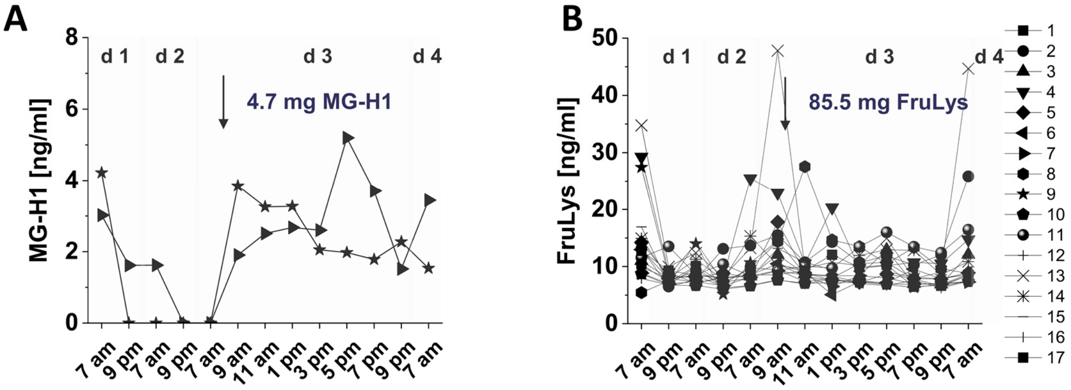

Figure S2. Impact of a high MRP intervention meal on salivary (**A**) MG-H1 and (**B**) FruLys concentrations during a three-days low MRP diet. Presented are the individual salivary of (**A**) MG-H1 and (**B**) FruLys concentrations for each of the (A: selected) subjects.

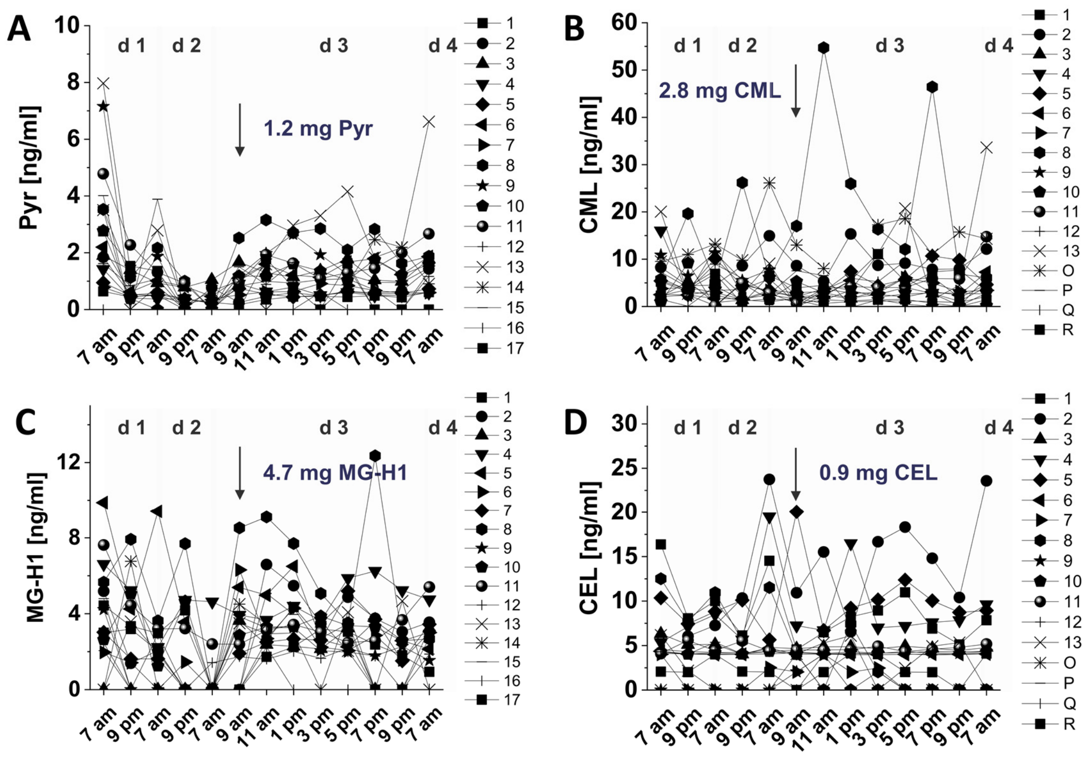

Figure S3. Impact of a high MRP intervention meal on salivary MRP concentrations during a three-days low MRP diet. Presented are the individual salivary (**A**) Pyr, (**B**) CML, (**C**) MG-H1 and (**D**) CEL concentrations for each of the subjects enrolled in the study.

Table S1. Raw data of Figure 1: Impact of dietary intake on salivary MRP concentration. "0" means no peak found during analysis, LOD means – peak height nearby the limit of detection.

Subject	d1 high	d2 high	d3 high	d4 high	d5 high	d1 low	d2 low	d3 low	d4 low	d5 low
					FruLys					
1	8.8	8.4	6.5	10.2	17.0	8.9	6.9	6.3	6.0	6.4
2	7.4	8.0	5.9	6.9	9.6	8.2	6.2	7.1	5.7	5.8
3	22.4	17.7	16.5	30.1	23.5	15.6	9.0	13.4	8.8	16.1
4	18.2	14.6	12.2	27.6	21.2	13.2	13.2	13.3	16.1	18.5
5	8.8	12.1	8.5	12.6	8.9	7.0	6.0	7.9	7.6	6.9
					MG-H1					
5	2.5	4.3	2.0	3.8	2.3	0.4	0	0	0	0
1	4.0	5.6	3.7	6.2	7.5	3.0	1.8	0.6	0	0
2	1.8	4.4	3.5	6.4	8.8	3.4	1.1	0	0	0
3	7.4	1.6	3.5	9.0	6.7	1.5	0.6	3.4	1.8	2.7
4	3.3	5.6	4.8	9.1	(1.6)LOD	4.1	0	0	0.4	0
					CEL					
1	5.2	3.5	4.8	5.1	9.7	4.9	6.1	4.9	5.2	4.9
2	6.2	6.0	3.7	4.2	7.7	3.2	2.4	4.3	4.3	5.1
3	18.0	17.3	10.9	11.7	17.9	11.1	6.2	9.9	7.0	8.7
4	7.2	6.0	2.8	7.3	6.1	2.5	9.8	8.6	13.3	10.7
5	6.8	13.8	8.4	17.6	9.9	5.3	4.9	6.9	2.4	6.3
					CML					
1	4.0	2.8	3.6	3.4	6.8	3.2	3.9	4.1	4.1	4.9
2	5.2	3.3	2.5	2.1	4.4	3.3	4.1	4.7	4.4	3.8
3	3.3	2.8	2.2	3.4	4.8	4.3	3.1	6.1	4.8	5.9

4	5.4	4.6	3.2	8.0	5.7	4.2	5.6	3.3	8.8	7.0
5	4.0	5.1	3.8	5.9	5.0	2.5	3.2	3.2	8.8	5.5
					Pyr					
1	3.4	3.3	3.1	3.4	4.0	3.3	3.1	0	0	0
2	3.1	3.2	3.1	3.3	3.4	3.2	0	0	0	0
3	3.3	3.5	3.9	4.1	3.9	3.1	0	0	0	0
4	3.5	3.5	3.5	4.0	5.0	3.2	3.1	0	3.3	3.4
5	3.7	3.4	3.4	3.5	3.1	3.2	0	0	0	0

Table S2. Raw data of the intervention study (Figures 3, 4 and S2, S3): Impact of a high MRP intervention meal on salivary MRP concentrations during a three-days low MRP diet. "0" means no peak found during analysis.

Subject	13	2	3	14	12	6	9	8	7	17	11	5	1	4	15	16	10
								CEL									
7:00 AM	16.4		6.3	5.4	10.4	4.4	4.0	12.5	0	4.3	4.1	4.5	6.2	0	4.3	4.1	2.1
9:00 PM	8.1	6.0	5.1	7.1	7.4	4.1	0	7.7	0	4.0	5.6	4.3	2.2	0	4.0	4.1	2.0
7:00 AM	10	7.3	5.1	8.5	8.8	4.0	0	11.0	0	4.2	4.4	4.2	0	0	5.4	4.0	4.0
9:00 PM	6.1	10.3	4.0	5.7	10.1	4.0	0	5.5	0	4.0	5.5	4.2	0	0	4.0	4.0	2.1
7:00 AM	14.5	23.7	4.8	19.5	5.7	4.7	2.5	11.5	0	4.3	4.4	4.3	0	0	4.1		2.0
9:00 AM	4.6	10.9	4.7	7.2	20.1	4.0	2.0	4.1	2.0	4.0	4.5	4.2	4.1	0	4.2	0	0
11:00 AM	6.5	15.5	4.9	6.7	6.7	4.1	4.0	0	4.0	0	4.5	4.1	5.3	0	4.1	4.0	2.0
1:00 PM	8.2	6.6	4.9	16.5	9.2	4.0	2.0	7.4	4.1	0	4.5	4.3	6.1		4.1	0	4.1
3:00 PM	8.9	16.7	4.7	7.0	10.1	4.0	2.5	2.0	4.1	0	4.9	4.2	0	0		2.1	4.0
5:00 PM	11.0	18.3	4.9	7.2	12.4	4.0	0	0	4.3	0	4.3	4.2	2.5	0	4.1	0	2.0
7:00 PM	6.9	14.8	4.7	7.6	10	4.0	0	0	4.2	0	4.6	4.3	4.9	0	4.0		2.0
9:00 PM	5.1	10.4	4.4	7.8	8.7	4.0	0	0	4.1	0	4.8	4.3	5.3	0	4.0	0	0
7:00 AM	7.8	23.6	4.8	9.6	8.9	4.1	0	0	4.3	0	5.2	4.4	0	0	4.2	0	4.1
								MG-H1									
7:00 AM	3.0	5.2	0	6.6	9.9	2.0	3.0	5.7	4.2	2.6	7.6	2.1	0	0	4.8	2.5	4.4
9:00 PM	3.2	1.4	0	5.2	4.3	1.4	1.6	7.9	0	5.0	4.4	0	3.6	6.8	0	0	3.3
7:00 AM	2.0	2.1	0	2.2	9.4	0	1.6	3.6	0	1.2	3.2	2.9	1.9	0	0	0	3.0
9:00 PM	0	0	0	4.7	3.6	1.5	0	7.7	0	4.7	3.2	0	0	0	0	0	4.2
7:00 AM	0	0	0	4.6	0	0	0	0	0	0	2.4	1.4	0	0	0		0
9:00 AM	0	0	3.6	3.9	5.4	6.3	1.9	8.5	3.8	2.9	2.4	1.7	3.5	4.5	0	0	0
11:00 AM	1.7	6.6	2.4	3.7	5.0	2.7	2.5	9.1	3.3	3.3	3.1	1.6	2.9	3.4	5.1	2.2	2.6
1:00 PM	2.3	5.5	2.3	4.4	6.5	4.3	2.7	7.7	3.3	3.3	3.4	2.0	3.0		4.0	0	4.3
3:00 PM	3.2	3.5	2.1	3.6	2.6	2.2	2.6	5.1	2.1	3.9	3.0	1.7	0	3.6		0	2.7
5:00 PM	3.0	4.9	3.2	5.9	2.3	2.1	5.2	3.5	2.0	3.4	2.5	2.2	4.1	2.0	2.6	0	3.5
7:00 PM	2.4	3.8	0	6.3	3.6	2.9	3.7	12.4	1.8	3.3	2.6	1.8	0	0	0		0
9:00 PM	1.8	3.0	0	5.2	2.6	1.6	1.5	2.6	2.3	2.6	3.7	0	4.7	0	0	0	0
7:00 AM	0.9	3.6	3.2	4.7	2.1	3.0	3.4	2.7	1.5	3.0	5.4	1.9	0	0	0	0	2.7
								FruLys									
7:00 AM	10.4	13.1	11.0	29.3	14.2	13.0	8.8	5.4	27.4	8.7	11.6	7.9	34.8	14.9	16.9	9.9	12.7
9:00 PM	7.3	6.5	7.9	9.0	8.3	7.9	7.0	7.5	8.5	6.8	13.5	6.8	10	7.9	6.8	7.9	9.3
7:00 AM	10.1	7.7	7.3	7.0	8.1	8.4	7.3	9.1	14.0	6.7	8.6	9.9	12.4	11.3	14.2	8.5	8.5
9:00 PM	7.1	13.1	8.0	8.1	8.9	8.1	6.6	7.0	5.1	6.0	10.4	6.2	8.2	7.7	7.3	6.5	8.1
7:00 AM	9.8	13.7	8.3	25.5	8.0	8.5	8.1	9.7	10.7	6.6	8.4	6.7	13.2	15.3	8.0		8.5
9:00 AM	9.4	15.5	12.1	22.9	17.8	10.5	9.2	14.4	14.5	7.6	9.3	7.7	47.8	10.2	13.2	10.5	8.6
11:00 AM	7.3	10.7	8.7	10.5	8.9	9.0	8.5	27.6	8.7	7.1	10.1	6.9	9.7	9.5	10.7	8.3	9.3
1:00 PM	8.0	14.3	8.4	20.3	7.6	5.1	6.5	14.7	8.3	7.2	9.7	7.9	10.8		9.4	7.6	12.1
3:00 PM	10.5	10.7	8.4	9.7	7.2	7.3	8.4	13.5	7.3	7.3	13.4	7.0	10.1	11.9		7.5	9.8

5:00 PM	10.7	11.8	12.9	10.2	8.7	7.2	8.0	10.5	6.8	6.9	16.0	6.7	14.9	12.9	9.1	7.1	10.3
7:00 PM	7.8	10.3	7.0	10.7	7.9	9.5	7.0	13.3	6.3	6.9	13.5	7.6	7.9	12.9	8.1		6.5
9:00 PM	6.6	12.0	6.9	10.5	7.8	7.9	6.9	9.6	6.9	7.0	12.4	6.2	9.0	8.6	7.9	6.4	6.7
7:00 AM	8.5	25.8	12.1	14.7	8.9	8.9	7.3	8.8	8.6	7.4	16.4	7.4	44.7	10.9	8.6	7.5	8.4
Pyr																	
7:00 AM	0.6	1.8	1.9	1.4	0.9	2.2	0.8	3.5	7.2	2.8	4.8	1.6	8.0	3.5	4.0	0	2.8
9:00 PM	0.4	0.5	1.4	0.4	0.5	0.6	0.5	1.1	0.5	0.4	2.3	0.8	1.4	0	0	0	1.5
7:00 AM	0.5	0.5	0.9	0.4	0.6	1.0	0.5	2.2	1.9	0	1.3	1.1	2.8	0.8	3.9	0	1.4
9:00 PM	0.4	0	0.8	0.4	0.4	0.8	0.2	1.0	0	0	0.9	0	0.1	0	0	0	0
7:00 AM	0.3	0.8	1.0	0.2	0.2	0.5	0.4	0	0	0	0	0	0.7	0	0		0
9:00 AM	0.2	0.3	1.7	0.9	0.7	1.1	0.5	2.5	1.2	1.2	0.9	0.8	1.0	0	1.5	0	0
11:00 AM	0.4	0.5	1.3	0.6	0.6	1.6	0.6	3.2	2.0	1.2	1.9	1.0	1.6	0	0.9	0	1.8
1:00 PM	0.5	0.6	1.1	0.8	0.4	1.2	0.6	2.7	2.6	1.6	1.6	0.9	3.0		1.7	0	0.8
3:00 PM	0.5	0.5	1.4	0.5	0.5	1.1	0.9	2.8	1.9	1.4	1.1	1.1	3.3	0		0.6	0
5:00 PM	0.4	0.6	1.8	0.6	0.6	1.7	0.8	2.1	1.2	1.0	1.3	1.1	4.2	0.9	1.5	0	1.6
7:00 PM	0.5	0.6	1.0	0.5	0.5	1.8	0.7	2.8	1.0	1.8	1.4	0.7	1.8	2.5	0		0
9:00 PM	0.4	0.7	1.0	0.5	0.6	1.2	0.6	1.6	0.9	1.2	2.0	0	2.2	2.2	0	0	0
7:00 AM	0.6	1.4	1.8	0.6	0.7	1.9	0.6	1.9	1.5	1.6	2.7	1.2	6.6	0.9	1.3	0	0
CML																	
7:00 AM	5.2	8.3	2.1	16.0	5.4	2.5	3.8	5.8	10.9	0.9	2.1	1.5	20.1	9.5	6.0	7.3	2.3
9:00 PM	3.2	4.4	5.3	2.5	3.6	2.4	4.0	19.6	6.5	9.2	2.4	0.1	2.7	11.0	3.4	4.0	3.6
7:00 AM	2.4	4.5	0.8	3.4	10.1	1.7	2.9	5.1	11.4	0.1	0.2	4.3	12.2	13.2	9.0	7.6	6.9
9:00 PM	1.2	8.6	1.4	3.6	4.5	1.6	1.5	26.2	5.5	4.9	5.0	1.4	2.7	9.8	6.0	2.3	0.6
7:00 AM	1.5	14.9	3.1	5.8	3.4	2.4	3.4	6.1	7.7	1.4	2.9	1.0	9.1	26.1	2.7		6.5
9:00 AM	1.3	8.6	2.0	0.6	2.9	1.6	2.1	17.0	2.2	5.3	0.6	1.8	0	13.0	7.5	0	3.9
11:00 AM	0.1	5.3	2.4	1.4	3.1	2.2	3.7	54.7	1.4	2.9	3.8	0.7	2.3	8.0	6.5	3.5	4.5
1:00 PM	0	15.3	1.7	3.5	7.4	5.5	2.9	26.0	1.2	1.8	4.2	1.1	5.6		0	6.0	3.4
3:00 PM	1.4	8.7	3.3	2.0	4.4	2.6	2.9	16.3	1.4	2.6	4.0	1.1	11.2	17.3		5.2	11.1
5:00 PM	1.5	9.2	6.1	2.6	5.7	2.0	6.1	12.1	0.5	4.0	4.0	1.2	20.8	18.6	4.2	9.6	5.3
7:00 PM	0.7	7.8	2.8	2.9	10.7	3.1	4.9	46.4	0	2.8	5.9	1.0	1.4	11.0	7.3		6.2
9:00 PM	0	8.0	1.1	2.8	9.8	2.5	3.2	6.9	0.1	0.2	5.8	0.9	4.5	15.8	7.7	0	1.9
7:00 AM	1.4	12.1	4.2	2.7	4.6	7.3	3.4	6.1	2.8	0.2	14.8	2.1	33.6	14.3	3.3	6.2	4.6

Table S3. Raw data of Figure 5: Concentrations of protein-bound FruLys and CML in the salivary protein fraction of five subjects during low or high MRP diet, related to lysine.

FL [µg/g Lys]	CML [µg/g Lys]	Subject	Dietary style
7.0	6.3	5	high
4.8	3.7	5	low
5.4	4.8	4	high
5.0	5.6	4	low
3.1	4.6	3	high
3.8	4.8	3	low
2.3	3.2	2	high
3.2	4.4	2	low
2.1	2.4	1	high
3.0	4.2	1	low
